# Risk Factors for Arterial Thromboembolic Events in Male Germ Cell Tumors Treated with Chemotherapy

**DOI:** 10.3390/cancers17142370

**Published:** 2025-07-17

**Authors:** Daniele Frisone, Melinda Charrier, Grégoire Berthod, Sara Manzocchi-Besson, Daniel Danzer, Sandro Anchisi, Petros Tsantoulis

**Affiliations:** 1 Service d’Oncologie, Département d’Oncologie, Hôpitaux Universitaire de Genève, 1205 Genève, Switzerland; melinda.charrier@hug.ch (M.C.);; 2 Service d’Oncologie, Centre Hospitalier du Valais Romand, Hôpital du Valais, 1951 Sion, Switzerland; gregoire.berthod@hopitalvs.ch (G.B.); sandro.anchisi@hopitalvs.ch (S.A.); 3 Service d’Angiologie et Hémostase, Centre Hospitalier du Valais Romand, Hôpital du Valais, 1951 Sion, Switzerland; sara.manzocchibesson@hopitalvs.ch; 4 Service d’Angiologie et Hémostase, Hôpitaux Universitaire de Genève, 1205 Genève, Switzerland; 5 Service de Chirurgie Vasculaire, Centre Hospitalier du Valais Romand, Hôpital du Valais, 1951 Sion, Switzerland; daniel.danzer@hopitalvs.ch; 6 Service de Chirurgie Vasculaire, Hôpitaux Universitaire de Genève, 1205 Genève, Switzerland; 7 Faculté de Médecine, Université de Genève, 1205 Genève, Switzerland

**Keywords:** testicular cancer, germ cell carcinoma, arterial thromboembolic events, smoking, thromboembolic events, risk factors

## Abstract

Background/Objectives: Germ cell tumors are the most common neoplasia in males < 50 y. In two case series, thromboembolic events (TEs) were reported in 8% and 13% of patients undergoing chemotherapy, whereas arterial thromboembolic events (ATEs) in other types of cancer treated with cisplatin had a frequency of 2% in a retrospective series and 0.67% in a meta-analysis. Recent data found a frequency of 2.4% for ATE in a large cohort of testicular cancer patients. Risk factors are not clearly identified, and given the severity of these events, further exploration is needed to determine appropriate preventive measures. Methods: We performed a retrospective cohort study of 171 patients undergoing chemotherapy for germ cell tumors in two centers in Switzerland and recorded the occurrence of ATE or venous thromboembolic events (VTEs) during chemotherapy or in the 3 months after its completion. Results: of 171 patients, 33.3% underwent adjuvant chemotherapy for stage I disease. Overall, 32 patients had a TE (18.7%, 95% CI 13.3–25.5%), 26 (15.2%, 95% CI 10.3–21.7%) had VTEs, and 11 (6.4%, 95% CI 3.4–11.5%) had ATEs. Five patients had both a VTE and ATE. VTEs were associated with disease stage (II, III, or relapse, with OR 15.6, *p* = 0.0002), retroperitoneal lymph nodes ≥ 3.5 cm (OR 3.2, *p* = 0.012), LDH > 500 UI/L (OR 5.3, *p* = 0.0025), and age > 35 y (OR 3.4, *p* = 0.005). The Khorana Score (KS) varied between 1 and 2 in 96% of the patients. ATEs were associated with active smoking (OR 6.5 *p* = 0.010), KS of ≥2 (OR 6.4 *p* = 0.004), and age > 35 y (OR 6.3, *p* = 0.01). Conclusions: Our findings show that ATEs are more frequent in our cohort than previous reports. We found a strong association between smoking and ATEs, which should be further assessed. Platinum-induced endothelial damage may be amplified by smoking in young patients in the absence of other risk factors and preventive medication.

## Simple Summary

Arterial thromboembolic events are considered a rare occurrence in young patients. In our study, we demonstrated an impressively high incidence of arterial thromboembolic events in patients undergoing chemotherapy for germ cell tumors, and we found for the first time a significant correlation with smoking status, older age, and Khorana score. These findings warrant prospective studies with the aim of improving the prevention of these potentially disastrous events.

## 1. Introduction

Cancer is a well-known thromboembolic risk factor. Thromboembolic events are associated with significant morbidity and mortality in cancer patients [1,2]. Among chemotherapeutic agents, cisplatin has been associated with an increased thromboembolic risk, up to 6.5-fold higher than in the general population [3,4].

Some cancer sites confer an important risk of thromboembolic events (TEs), as assessed by Khorana et al. during the validation of a thromboembolic risk model in patients with cancer in a retrospective cohort of 2701 patients, validated in a prospective cohort of 1365 patients [5]. In this model, pancreatic and gastric cancer confer the highest risk (“very high”) of TEs, scoring 2 points, while other sites of cancer such as lymphoma, testicular, and lung each score 1 point (“high risk”).

The Khorana Score (KS) did not specifically investigate arterial thromboembolic events (ATEs), which are associated with considerable morbidity. ATEs seem to be more frequent in cancer patients but are not necessarily associated with cisplatin-based chemotherapy, although this has been poorly investigated [6,7,8,9]. Few studies have specifically focused on ATEs, including a retrospective study on germ cell tumors including 179 patients treated with chemotherapy. Fifteen patients (8.4%) developed major TEs, of whom three patients developed ATEs (1.7%) [10]. In a Norwegian cohort of 506 patients, the prevalence of ATEs was 2.4%, while the prevalence of VTEs was 11.5%. In this study, the use of thromboprophylaxis did not seem to reduce the incidence of VTEs [11]. Even if historical data seem to indicate an ATE incidence of less than 1% in unselected patients undergoing cisplatin chemotherapy, no direct comparison exists between germ cell and non-germ cell tumors patients.

In our daily practice, we were puzzled by recurrent cases of ATEs in germ cell tumor patients, especially those who smoked, which led us to perform a retrospective analysis with the aim of better characterizing the potential risk factors for TEs, specifically for ATEs. We hereby present the results of a cohort of 171 patients treated in two centers in Switzerland between 2010 and 31 May 2023.

## 2. Materials and Methods

We performed a retrospective analysis on a cohort of male patients treated and followed for germ cell tumors in the Geneva University Hospitals (Geneva, Switzerland) and the Valais Hospital (Sion, Switzerland). Included patients were diagnosed with germ cell tumors and underwent chemotherapy as either adjuvant, first-line treatment, or at first relapse after the initial surveillance strategy.

TEs were identified and considered only when occurring in the period between the initiation of chemotherapy and the 3 months following the last cycle of systemic treatment. This permitted us to include and evaluate all patients with possible acute toxicities of systemic chemotherapy, excluding patients with late onset cardiovascular toxicity. ATEs were defined according to international clinical practice as objectively confirmed myocardial infarction, stroke, central or peripheral arterial thrombosis, or sudden death. This was intended to not include patients with clinical suspicion of other types of arterial embolism (air, fat, septic, or neoplastic) if encountered, but no patient met such criteria. VTEs were defined as deep venous thrombosis and/or pulmonary embolism.

All patients had a documented follow-up control visit at least three months post-chemotherapy, and most patients had been followed in the two institutions according to EAU guidelines (with scheduled visits every three months for the first two years at least).

Based on information recorded in the electronic or paper hospital database, we collected additional variables including age, histology, tumor stage, chemotherapeutic regimen (BEP, carboplatin, or other platinum-based protocols), maximum diameter of retroperitoneal lymph-nodes, active tobacco smoking (<1 year), Body Mass Index (BMI), Khorana score before chemotherapy initiation, ongoing treatment with antiplatelet (aspirin, P2Y12 inhibitors), anticoagulant (LWMH, DOA, anti-vitamin K), or hypolipemiant drugs, history of hypertension or diabetes, and LDH concentration. A few patients received prophylactic dose anticoagulant at the clinician’s discretion at chemotherapy initiation.

Since tumor stage is reported at diagnosis, we used the term “localized” for stage I disease to indicate true adjuvant chemotherapy. Cases initially diagnosed as stage I but included at relapse are indicated as extensive disease, as well as stages IS, IIA-III. Following current recommendations, IGCCCG classification was applied for stages IS, IIC, or III or for relapsed disease.

As in previously published studies, and because the timing of endothelial recovery from smoking-associated dysfunction is not precisely known, we defined active smoking as smoking cessation within at least a one-year interval [12,13].

All data were collected using the information recorded in the electronic hospital database. The first and last co-authors were responsible for data collection in the two sites.

Statistical analyses were performed with the R language for statistics (version 4.3.1). Fisher’s exact test was used to test the association between potential risk factors for the development of ATEs or VTEs and the occurrence of an event. We also performed logistic regression for multivariable analyses against the occurrence of an ATE or VTE. The primary analysis included the association of smoking, hypertension, diabetes, disease extension, LDH value, Khorana score, chemotherapy type, histology, enlarged retroperitoneal lymph nodes (>3.5 cm), and age with ATEs, VTEs, or both.

The study protocol was approved by the local Ethical Committee (CER-VD 2021-00749). This research did not receive any specific grant from funding agencies in the public, commercial, or not-for-profit sectors.

## 3. Results

Our study included a total of 171 patients from two centers in Switzerland (106 Geneva, 65 Sion) treated from January 2010 to May 2023. Of the 183 patients identified to be eligible to our study, 10 patients did not consent. Of the remaining 173, two patients were excluded since follow-up data were not available.

A total of 57 patients had stage I disease (localized) and received adjuvant chemotherapy. As shown in Table 1, less than 6% of the patients had known diabetes, hypertension, or were already on antiaggregant/anticoagulant treatment, while 41.5% of all patients were active smokers. Concerning thromboembolic risk, 76% of the patients had a KS of 1, 19.8% scored 2, while only 7 patients (4%) had a KS of 3 or more.

### 3.1. Events

**Figure 1:**
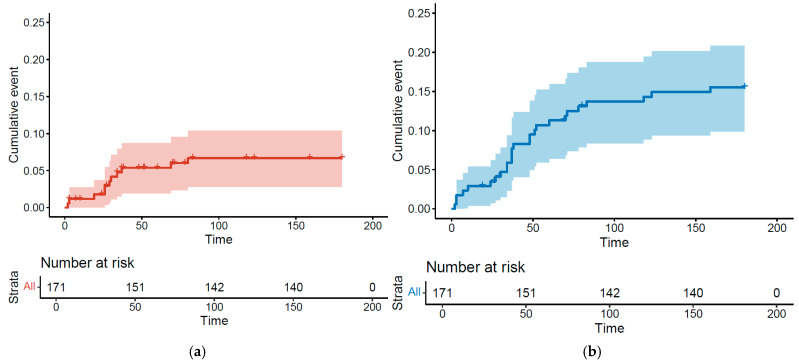
Kaplan–Meier estimates for cumulative incidence of arterial thrombosis (**a**) and venous thrombosis (**b**).

Overall, 32 patients (18.7%) had a thromboembolic event during chemotherapy or in the 3 following months. We identified 26 VTEs (15.2%) and 11 ATEs (6.4%), including 5 patients (2.9%) who had both a VTE and ATE. Kaplan–Meier curves illustrate the cumulative incidence for these events and show that VTEs and ATEs mostly occur within the first two chemotherapy cycles (Figure 1a,b).

### 3.2. Risk Factor Analysis

#### Venous Thromboembolic Events

As expected, VTEs were more frequent in patients with advanced disease (25 events, OR 15.6 versus localized disease, *p* = 0.0002). Only one event occurred in the 57 patients undergoing adjuvant chemotherapy post-orchidectomy for localized disease. Larger retroperitoneal lymph nodes were associated with VTEs (OR 3.2 for patients with LN > 3.5 cm, *p* = 0.012). Age over 35 y was an additional risk factor (OR 3.4, *p* = 0.005), as well as LDH level > 500 UI/L (OR 5.3, *p* = 0.0025). Interestingly, the KS was not associated with higher TVP risk, nor was smoking status, high blood pressure, or histological subtype.

### 3.3. Arterial Thromboembolic Events

ATEs were significantly associated with smoking status (OR 6.5, *p* = 0.010), KS of 2 or more (OR 6.4, *p* = 0.004), and age > 35 y (OR 6.3, *p* = 0.011). There was a statistical trend favoring ATEs in patients with “extensive disease” (OR 5.3. *p* = 0.10) or elevated LDH (OR 3.4, *p* = 0.10), while no association was found with chemotherapy protocol (especially cisplatin vs. carboplatin, OR 2.12, *p* = 0.69), histology, or high blood pressure.

**Figure 2:**
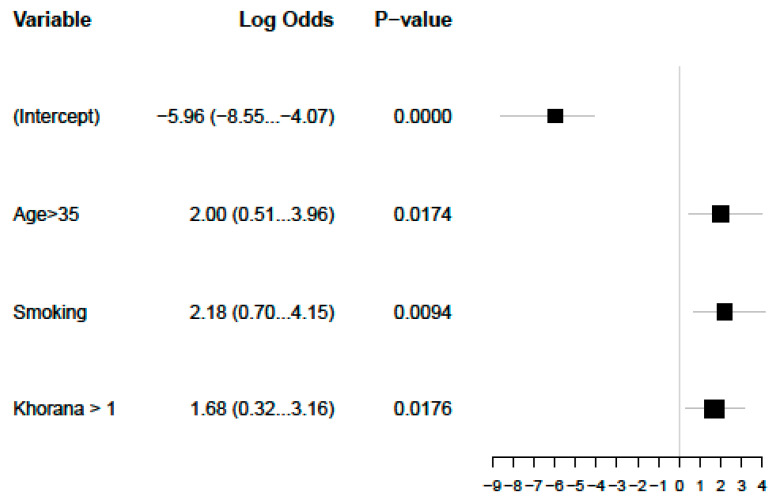
Significant multivariate analysis for ATEs.

**Figure 3:**
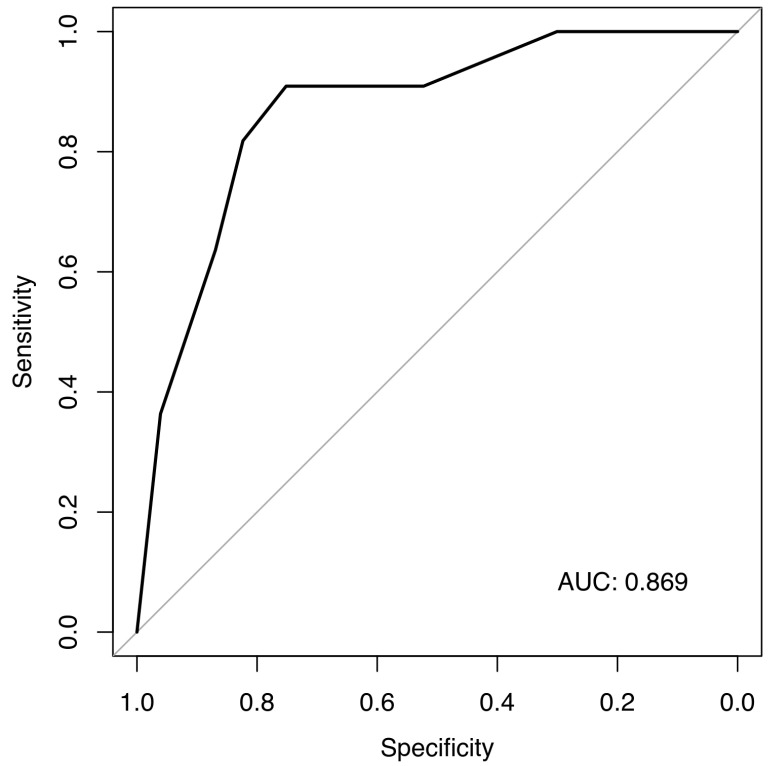
ROC for our prediction model for ATEs.

We performed a multivariate analysis that confirmed smoking, KS, and age as predictors of ATE in patients with germ cell carcinomas (Figure 2). A model using these three simple items fits our data well, with an area under the curve of 0.869, although sensitivity and specificity will have to be estimated in a separate validation dataset (Figure 3).

Characteristics of patients undergoing an ATE are described in Table 2.

## 4. Discussion

Despite a small number of events, our data on ATEs should raise concern, as we found a surprisingly high incidence of these events in young patients treated with platinum-based combinations, together with a strong and significant correlation with active smoking, KS, and age. This high rate of cardiovascular events has been described in a Danish study, which demonstrated a 24-fold higher risk of VTEs but also a 6-fold higher risk of myocardial infarction in the first year from the beginning of BEP treatment [14]. Our data confirm and increase knowledge about this topic, being in line with the initial findings of a Norwegian cohort study conducted by Haugnes and colleagues [11].

We do not give a simple or unequivocal explanation for our findings, especially the high incidence of ATEs in our cohort. We found an impressively high prevalence of people who smoked in our cohort, and it is known that smoking is a possible risk factor for testicular cancer, even if its role is controversial [15].

Moreover, smoking habits have a different trend in Switzerland compared to other countries such as UK, with one-quarter of the adult population reported to smoke in a recent epidemiological study [16,17].

A recent publication has defined a model predicting the risk of TEs using a simple tool analyzing smoking status, weight, hypertension, hypercholesterolemia, and diabetes at baseline (called vascular fingerprint). The model was developed in 196 patients with metastatic germ cell tumors of IGCCCG good or intermediate risk and a retroperitoneal mass of less than 5 cm. The presence of high risk (three risk factors) conferred a three times higher risk of TEs, especially ATEs. In this prospective study, no correlation with smoking status as an independent risk factor for TEs was found, but only 4 ATE cases were reported [18].

In our cohort, we found that active smoking and high Khorana score were associated with ATEs in relatively young men without other risk factors. A model tested with our data achieved an excellent fit, although its sensitivity and specificity need to be validated in a larger dataset. A specific predictor for ATEs in this context could help identify patients at risk and propose a more proactive type of surveillance and prophylaxis.

We tried to reproduce the “vascular fingerprint model” on our dataset, although analysis of LDL cholesterol or Hb1Ac had not been systematically performed at baseline. Only anamnestic or clinical data on diabetes, hypercholesterolemia, or hypertension were collected. Interestingly, we found a different distribution of risk assessment, with only 4% of patients in our cohort having a high-risk vascular fingerprint. The OR for ATE was estimated at 2.4 and was not significant (*p* = 0.39).

The definition of active smoking can vary in literature and depending on the underlying involved mechanism, which may have differential impacts on TE risk. Here, active smoking was defined as ongoing or having stopped less than a year from chemotherapy initiation and was significantly associated with ATEs. Of the 11 patients who developed ATEs, only 1 patient quit smoking in the year before chemotherapy, the others still being smokers at the moment of diagnosis. We decided to perform a sensitivity analysis considering this patient as a “non-smoker”. The univariate analysis showed an OR of 3.9 (*p* = 0.056), and the multivariate analysis showed an association that remained significant (*p* = 0.03).

Finally, a very important question is whether prophylactic anticoagulation is useful in this population. In our cohort, only nine patients received prophylactic anticoagulation (9/171, 5.3%), and two of these nine patients had a TE. Given these small numbers, no association was found, and no conclusions on this type of practice can be made in the absence of randomization, considering that patients were selected for prophylactic anticoagulation based on the physician’s estimation of their individual risk.

For ATEs, instead, prophylactic anticoagulation is still a matter of debate in this specific context, and it is difficult to decide based only on observational data. A recent review and meta-analysis of 14 randomized studies on this subject did not show a significant effect for this strategy, using either LMWH or new direct oral anticoagulants [19]. The role of antiplatelet drugs such as aspirin in primary prevention is even less clear, given the absence of prospective studies [20].

**Figure 4:**
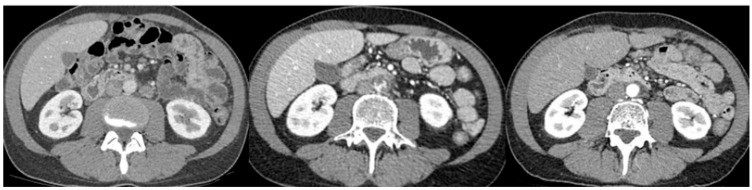
Appearance of aortic and mesenteric artery thrombus during chemotherapy and disappearance with therapeutic anticoagulation at first follow-up.

Interestingly, most patients suffering from ATEs in our cohort who underwent a comprehensive angiologic workup did not have underlying atherosclerotic lesions. One study based on a German database focused on ATEs in testicular cancer but did not find such an incidence and could not identify risk factors for ATEs in the cohort. In this study, similarly, patients with myocardial infarction did not have clear coronary artery disease [21]. This repeated observation in a young population raises questions about its cause, as knowing the pathogenetic mechanisms is the first step that could lead to treatment and prevention of this rare but extremely morbid event. We present here, as an example, a contrast-enhanced CT scan of a 44-year-old patient experiencing acute mesenteric ischemia and a visible intra-aortic and upper mesenteric artery thrombus, occurring during chemotherapy in the absence of underlying atherosclerotic lesions before and after the chemotherapy period (Figure 4).

Considering VTEs, the results from our analysis are consistent with previous reports on VTE risk in germ cell tumors [22]. The incidence of thrombotic events in our study was high, raising the concern that many of these events could remain undetected in the absence of appropriate tests [23]. Known risk factors for VTEs investigated in larger trials, such as RPLN diameter > 3.5 cm, were also found to increase risk in our study [22].

Remarkably, the KS was below 3 for 96% of our patients. The KS does not differentiate between stage I and stage II/III tumors, which seem to have a much higher risk of VTEs. In this sense, stage II–III or relapsed germ cell carcinoma would seem to belong in a category of very high risk, as the incidence of VTEs in this population seems to be over 10% in all recent studies, including ours (15.2%). This question was also raised in a recent Swiss consensus recommendation, with half of the experts suggesting the use of prophylactic anticoagulation for metastatic germ cell tumors undergoing chemotherapy, regardless of other risk factors [24].

The physiopathology of cisplatin-associated vascular damage leading to thrombosis is not well understood. Possible explanations, such as endothelial dysfunction, platelet activation together with a rise of Von Willebrand factor, and hypomagnesemia-induced vasospasm, have been discussed [6,25,26].

A recent work highlighted once more the important role of endothelial dysfunction in cisplatin-associated vascular damage, focusing on testicular cancer patients. This work, analyzing different surrogate markers of endothelial dysfunction and blood metabolites, found acute changes in endothelial function and lipid and Hb1Ac levels throughout the first weeks of treatment with BEP [27].

Another insight for further research concerns the possibility of a pro-thombogenic molecular landscape, as suggested by recent work affirming that KRAS mutation and STK-11 are associated significantly with ATEs, independently of tumor type, even if the mechanism for this association has not been clarified [28]. KRAS mutations are present in germ cell tumors (12%) while STK11 are rare, found in less than 1% of mutations identified based on TCGA analysis [29]. Further molecular analyses, currently unavailable in our cohort, may be able to test this hypothesis.

Finally, it is important to mention that when treating germ cell tumors, attention to cardiovascular disease prevention remains a goal in survivorship care, as an increase in the rate of cardiovascular diseases has been clearly described in cancer survivors, especially after cisplatin-based chemotherapy [30]. Chronic vascular damage seems to be the result of cisplatin-induced endothelial dysfunction, with a higher risk of developing metabolic syndrome, hypertension, and hypercholesterolemia [31].

## 5. Conclusions

Our study highlights the high incidence of thromboembolic events in patients with germ cell tumors undergoing chemotherapy while also recognizing, for the first time, an association between arterial TE and smoking status. A simple model including KS, smoking habit, and age >35 fits well in our data and could be prospectively validated with larger-population studies. Other cardiovascular risk factors such as hypertension, hypercholesterolemia, and diabetes had a very low prevalence in our dataset, but they were not actively searched in the baseline work-up. Further research, especially on tumor molecular features and host genetics, may help to explain the occurrence of these potentially disastrous events.

## Figures and Tables

**Table 1:** Baseline patient characteristics, N (%).

Seminoma	78 (45.6)
Non-seminoma	93 (54.4)
Initial disease stage	
I	74 (43.2)
II	42 (24.6)
III	55 (32.2)
Localized vs. extensive * (chemotherapy indication)	
Localized (adjuvant, no macroscopic residual disease)	57 (33.3)
Extensive (curative, macroscopic residual disease)	114 (66.7)
IGCCCG classification	
Good	57 (33.3)
Intermediate	9 (5.3)
Poor	14 (8.2)
Not applicable (stage I or IIA,B)	91 (53.2)
Chemotherapy type	
Carboplatin AUC7	30 (17.5)
BEP	117 (68.4)
EP	17 (9.9)
Others **	7(4.1)
Active smoker ***	
Yes	71 (43.3)
No	93 (56.7)
Diabetes	4 (2.3)
Hypertension	10 (5.8)
Ongoing anticoagulation	9 (5.3)
Ongoing antiplatelet	4 (2.3)
Ongoing hypolipemiant	3 (1.8)
Khorana score	
1	130 (76)
2	34 (20)
3 or more	7 (4)
Median diameter of retroperitoneal lymph nodes (cm)	2.77 (0–22)
LDH (median)	207 (107–7380)
Age > 35 y	75 (43.9)
Age—median	35 (range 18–66)
BMI—median	24.8 (range 15–48)

* Extensive: includes any recurrence after initial stage I, stage II, III, and IS. ** Cisplatin Etoposide Ifosfamide (4), Bleomycin Carboplatin Etoposide (1), Ifosfamide Carboplatin Etoposide (1), Cisplatin Etoposide Mitoxantrone Cytarabine (1). *** includes patients who stopped smoking less than a year before.

**Table 2:** Characteristics of patients experiencing ATE.

Birth Date	Age at Diagnosis	Type of Tumor	Stage at Instauration of Chemotherapy	IGCCCG	KS	Smoking Status	HTA	Type of Chemo	Type of ATE	VTE Associated	Treatment of ATE Complementary to Anticoagulation	Complementary Information
1972	48	Seminoma	III (TxN2M1a)	Good	1	no		3xBEP	Acute left humeral-radial and ulnar arterial thrombosis—subacute left renal ischemia (infarction at the lower third)	APE	Surgical thrombo-embolectomy of humeral artery	Transthoracic and transoesophagial echocardiography normal, no AF—no dysrhythmia on Holter.
1970	47	Seminoma	IB (pT3 cN0M0)		1	Yes (35 UPA)		1 Carboplatine AUC7	STEMI	-	Coronary stenting	Diabetes Obesity (BMI 33) Hypeholesterolemia
1978	40	Seminoma	III (pT3N1M1a) S0	Good	1	Yes (15 UPA)		4xEP	Acute right popliteal and posterior tibial artery occlusion	-	Surgical thrombectomy	Obesity (BMI 32)
1971	48	Seminoma	Relapsing (pulmonary metastasis)	Good	2	Yes (30 UPA)		4xEP	Splenic arterial thrombosis with infarction	PE		
1974	45	Seminoma	Relapsing IIB S0	Good	2	Yes (22 UPA)		4xEP	Acute superior mesenteric artery occlusion	-	Ileal resection	
1970	42	NSGCT	IIIB (pT2cN2cM1b) S2	Intermediate	3	Yes (26 UPA)		4xEP	STEMI	PE left Renal vein thrombosis		Right coronary thrombosis
1962	60	Seminoma	pTxcN2M1aS0	Good	1	No (stopped 15 year before but 48UPA)		4xEP	STEMI	-	Angioplasty and stent IVA	
1951	66	Seminoma	III (TxN3M1) S1	Good	2	Yes (40 UPA)	Yes	3xEP	Acute thrombus of the deep femoral artery	-	Surgical endarterectomy and thrombectomy	Background of pre-existing lower limbs arteriopathy
1994	23	NSGCT	III (TxNxM1) S3	Poor	2	Yes (intermittent)		VIP	Radial artery thrombosis	Subclavian vein DVT		
1985	34	NSGCT	III (TxNxM1) S1	Poor	3	Yes (14 UPA)		4xBEP	Frontal ischemic stroke	Jugulo-subclavian and innominate vein DVT		Previous STEMI Familial hypercholesteremia Obesity (BMI 32)
1973	41	Seminoma	Relapsed IIB S0	Good	3	Yes (60 UPA)		4xBEP	Sudden death	-		Obesity (BMI 46)

One case of cerebral ischemic attack was attributed to a VTE with patent foramen ovale.

## Data Availability

The raw data supporting the conclusions of this article will be made available by the authors upon request.

## Abbreviations

TE: Thromboembolic events. ATE: Arterial thromboembolic events. VTE: Venous thromboembolic events.
